# Field Evidence of Cadmium Phytoavailability Decreased Effectively by Rape Straw and/or Red Mud with Zinc Sulphate in a Cd-Contaminated Calcareous Soil

**DOI:** 10.1371/journal.pone.0109967

**Published:** 2014-10-10

**Authors:** Bo Li, Junxing Yang, Dongpu Wei, Shibao Chen, Jumei Li, Yibing Ma

**Affiliations:** 1 National Soil Fertility and Fertilizer Effects Long-term Monitoring Network, Institute of Agricultural Resources and Regional Planning, Chinese Academy of Agricultural Sciences, Beijing, P. R. China; 2 Institute of Plant Nutrition and Environmental Resources, Liaoning Academy of Agricultural Sciences, Shenyang, P. R. China; 3 Centre for Environmental Remediation, Institute of Geographic Sciences and Natural Resources Research, Chinese Academy of Sciences, Beijing, P. R. China; Chinese Academy of Sciences, China

## Abstract

To reduce Cd phytoavailability in calcareous soils, the effects of soil amendments of red mud, rape straw, and corn straw in combination with zinc fertilization on Cd extractability and phytoavailability to spinach, tomato, Chinese cabbage and radish were investigated in a calcareous soil with added Cd at 1.5 mg kg^−1^. The results showed that water soluble and exchangeable Cd in soils was significantly decreased by the amendments themselves from 26% to 70%, which resulted in marked decrease by approximately from 34% to 77% in Cd concentration in vegetables. The amendments plus Zn fertilization further decreased the Cd concentration in vegetables. Also cruciferous rape straw was more effective than gramineous corn straw. In all treatments, rape straw plus red mud combined with Zn fertilization was most effective in decreasing Cd phytoavailability in soils, and it is potential to be an efficient and cost-effective measure to ensure food safety for vegetable production in mildly Cd-contaminated calcareous soils.

## Introduction

Recently, increasing cadmium (Cd) accumulation in vegetables is a growing concern globally because of increased fertilizer- and biosolids-borne Cd in soils [1]–[3]. As a consequence, international trade organizations have sought to limit the concentration of Cd in some crops sold in international markets. The National Food Hygiene Standard of China (NFHSC, GB 15201-94) proposed maximum levels of 0.05 mg kg^−1^ of Cd for spinach and other vegetables. However, there are some areas where Cd concentrations in vegetables are over the limit [4], [5]. Therefore, reduction of Cd uptake by vegetables and translocation to edible parts is one of the important strategies for proper use of mildly Cd-contaminated soils and safeguarding the safety of farm produce [1].

Nowadays, “in situ” remediation techniques of mildly Cd-contaminated soils are regarded as possible effective approaches to address the issues of excessive vegetable Cd concentrations. During the last decade, the possibility of Cd immobilization in soils through the addition of different amendments or sorbent, has been extensively investigated in order to reduce the risk of groundwater contamination, plant uptake, and exposure to living organisms [6]–[9]. Among these amendments or sorbents, red mud (RM), a by-product of aluminium (Al) manufacturing, can be very effective in increasing Cd sorption and decreasing soluble Cd concentrations in Cd-contaminated and acidic soils under pot trials [10]–[13] and field studies [14], [15], and lead to a reduction in Cd uptake by plants. Lombi et al. [16] indicated that the specific sorption of Cd by Fe and Al oxides in RM was the main mechanisms of fixation. It is therefore important to select cost-effective and feasible amendments to immobilize Cd by specific sorption. It has been documented that thiol (-SH) can reduce Cd bioavailabilty by mechanisms of chelation [17], [18]. The cruciferous rape (Brassica napus L.) exhibited higher concentration of thiol (-SH) in straw than other crops [19], [20]. However, the effect of the incorporation of rape straw (RS) into Cd-contaminated soil has never been investigated under field condition. In addition, use of zinc (Zn) fertilizers in soils, such as ZnSO_4_, has been reported to decrease the accumulation of Cd in crops [21]–[24]. Abdel-Sabour et al. [21] reported that the Cd/Zn ratio in plant tops was significantly affected by both Cd and Zn concentrations in soil. Yang et al. [23] also found that the application of foliar Zn or seed Zn fertilizer could significantly decrease the Cd concentration in cucumber shoots by about 12–36% in Cd-contaminated soils. Köleli et al. [24] also expressed that Cd toxicity in the shoots of bread and durum wheat was alleviated by Zn treatment.

Therefore, we hypothesized that the Cd immobilizing amendments of RS plus RM in combination with Zn fertilizer might be more effective in reducing Cd accumulation in vegetables grown in Cd-contaminated and calcareous (high pH) soils under field conditions. The present study was conducted to investigate the efficiency of these amendments with Zn fertilization on Cd accumulation in the edible parts of four common vegetables (spinach, tomato, Chinese cabbage and radish) grown in mildly Cd-contaminated and calcareous soil under field condition. Furthermore, the effect of Cd immobilizing amendments (RS, RM, RS+RM) with Zn fertilizer on the Cd fractions associated with different soil components was also studied. The results will be helpful to find practical and cost-effective measures to reduce Cd accumulation in crops.

## Materials and Methods

### Soil characteristics and amendments used

The field experiment was conducted at long-term experiment station of the Chinese Academy of Agricultural Sciences, Dezhou (DZ) city, Shandong Province, China (37°20′N, 116°29′E). The soil used in the field experiment had a pH (1∶5 soil/water suspension) of 8.9 and contained 1.2% organic matter, 6.17% CaCO_3_, 0.08% total N and 0.1% total P as measured by the standard methods given in Jackson [25]. The soil contained 64% sand, 18% clay and 18% silt. Total Zn and Cd concentrations of the soil were 54 mg Zn kg^−1^ and 0.11 mg Cd kg^−1^, measured as described by Jackson [25], while DTPA-extractable [26] concentrations of Zn and Cd were 0.11 mg Zn kg^−1^ and <0.005 mg Cd kg^−1^, respectively.

Red mud, RS, and corn straw (CS) were used as immobilizing amendments in the field experiment. Red mud (pH = 11.1) was from Zibo City, Shandong Province, China. The mineralogical composition of RM sample (XRD analysis) is a mixture of SiO_2_ (20%), Fe_2_O_3_ (28%), Al_2_O_3_ (21%), CaO (6.2%), MgO (1.3%), TiO_2_ (3.3%), K_2_O (0.26%) and Na_2_O (11%). The specific surface area, determined by the BET/N_2_-adsorption method (Sorptomatic CarloErba), was 12.2 m^2^ g^−1^ for RM. Zinc and Cd concentrations in RM were 94 mg kg^−1^ and <0.01 mg Cd kg^−1^, respectively. The RM sample was dried overnight at 105°C, finely ground and sieved to <1 mm. The rape straw sample was obtained from rape (Allium cepa L. cv. Zheshuang No. 6) grown at long-term experiment station of the Chinese Academy of Agricultural Sciences, Jiaxing city, Zhejiang Province, China (30°15′N, 120°20′E), which was oven-dried at 70°C to constant weight and then finely ground in a Retsch-grinder (Type: 1 mm, made in Germany) using a 1 mm mesh screen to ensure uniform plant tissue disruption and distribution in soil during the field experiment. The rape straw with pH of 6.41 and electrical conductivity (EC) of 398 µS cm^−1^ (straw:solution ratio 1∶10), contained 19 mg Cu kg^−1^, 23 mg Zn kg^−1^, 0.86 mg Pb kg^−1^ and 0.67 mg Cd kg^−1^ dry weight. The corn straw sample was obtained from corn (Zea mays L. cv. Jingdan No. 28) grown at long-term experiment station of the Chinese Academy of Agricultural Sciences, Changping, Beijing, China (40°13′N 116°15′E). The sample was also oven-dried at 70°C to constant weight and then finely ground using a 1 mm mesh. The corn straw with pH of 6.10 and EC of 187 µS cm^−1^, contained 36 mg Cu kg^−1^, 59 mg Zn kg^−1^, 0.76 mg Pb kg^−1^ and 0.84 mg Cd kg^−1^ dry weight.

### Field experiment and plant analysis

The field experiment was a randomized complete block split-spot design with 3 replications for the control and amendment treatments (main treatments) and 2 replications for the control and amendment treatments (sub-treatments) in combination with Zn fertilization (12 g ZnSO_4_ per plot, based on the mass of top 20 cm soil). The size of each plot was 4 m^2^ (2 m×2 m). Before amendment addition, soils in the plots were added with 1.5 mg Cd kg^−1^ in the form of CdSO_4_ on 1 February, 2009. The concentration of Cd added to soil was chosen based on preliminary experiments and represented mildly Cd contamination. To decrease the variability, the salts of CdSO_4_ were mixed with topsoil samples (0–20 cm) separately in a container, after which the spiked soils were returned to the plots and equilibrating for 2 months. Fertilizers were then applied to all plots according to local farming practices. The equivalent nitrogen (0.2 g N kg^−1^ soil as urea), phosphorus (0.06 g P_2_O_5_ kg^−1^ soil as superphosphate), potassium (0.06 g K_2_O kg^−1^ soil as potassium sulfate) were applied as basal fertilizers to each plot before the spinach and tomato seeding. After spinach and tomato harvest, the same equivalent phosphorus, potassium and nitrogen were applied as basal fertilizers to each plot before Chinese cabbage and radish seeding. All nutrients were mixed homogenously with soil before sowing.

The main treatments were (1) control, (2) 0.5% RM (W/W), (3) 0.1% RS (W/W), (4) 0.5% RM+0.1% RS, (5) 0.1% CS (W/W), (6) 0.5% RM+0.1% CS. The sub-treatments were applied with Zn fertilization (12 g ZnSO_4_ per plot) before the Cd-contaminated soils in the plots had been added with the amendments mentioned above except CS treatment. The amendments were applied to the surface of each plot before being ploughed into the soil to a depth of 20 cm.

Four commonly cultivated vegetable varieties (Spinach (Spinacia oleracea L. cv. Huabo No. 1), Tomato (Lycopersicum esculentum Mill. cv. Lufen No. 3), Chinese cabbage (Brassica campestris L. cv. Degao No. 16) and Radish (Raphanus sativus Linn. cv. Qianxi No. 2) in DZ were selected in this experiment and were sown directly into the soil according to different growth periods. Spinach was sown on 3 April and harvested on 10 May, 2009. Tomato was sown on 5 May, and harvested on 15 September, 2009. Radish and Chinese cabbage were sown simultaneously on 16 October, 2009 after harvest of spinach and tomato. Sufficient seed was sown to guarantee healthy germination, then seedlings were thinned after germination). Only the edible portions were sampled after all vegetables were grown to maturity as the study was focused on the food safety. At each plot, 10 subsamples of the edible parts of vegetables were collected and combined for chemical analysis. The fresh vegetable samples were put in clean plastic bags and transported to the laboratory for sample treatment. The samples were washed with 0.2% HCl solution followed by tap water and de-ionized water, then oven-dried (not peeled) at 70°C for 6 h to constant weight and dry weights (DW) were recorded. The plant samples were ground using a Retsch-grinder (Type: 0.5 mm, made in Germany), then weighted 0.5 g to 200 mL digestion tubes with 10 ml of concentrated nitric acid (HNO_3_) and digested for 9 h at 110°C after standing overnight [27]. Cadmium concentrations were determined using inductively coupled plasma mass spectrometry (ICP-MS). Blank and bush leaf material (BGW-07603) (China Standard Materials Research Center, Beijing, PR China) were used for quality control. The Cd recovery rates were 90±10%.

### Soil analysis by sequential extraction procedure

After harvest, 10 subsamples of soils (0–20 cm) were evenly collected from each plot, bulked together, air-dried, and ground to pass a 0.26-mm sieve. Soil pH was measured using de-ionized water (1∶5 soil/water suspension) with an ORION combined electrode. The fractions of Cd bound to the soil were determined by a sequential extraction procedure according to Basta and Gradwohl [28], in order to study the effects of the different amendments on Cd fractions. To extract the water soluble fraction (WS-Cd), each sample collected from plots (2 g) was treated with 25 mL of de-ionized water (pH 6.5) and shaken for 2 h at room temperature. It was then treated with 25 mL of 0.1 N Ca(NO_3_)_2_ solution to extract the exchangeable fraction (Exch-Cd), and with 25 mL 0.02 M EDTA solution to extract the complexed fraction (EDTA-Cd). After each step of the extraction process the samples were centrifuged at 10,000 rpm for 0.5 h and filtered to separate the liquid and solid phases. After the third extraction, the residual form of Cd (Res-Cd) was determined by drying the solid phase overnight at 105°C and digesting it with HNO_3_ and HCl (ratio 1∶3) in a Microwave Milestone MLS 1200. The Cd concentrations in each extract or digest were determined using inductively coupled plasma mass spectrometry (ICP-MS).

### Data analysis

All results were presented as arithmetic means with standard errors and analyzed by SPSS 11.0 statistical package. Statistical comparisons of means of plant data were analyzed with one way ANOVA followed by the Fisher’s least significant test. Correlation coefficient analyses were conducted using program of Origin 7.0.

## Results

### Effects on Cd in vegetables

The concentrations of Cd and yield for edible parts of spinach, tomato, Chinese cabbage and radish with both the unamended and amended soil combined with Zn fertilization are presented in Table 1. Compared with the vegetable grown in unamended soil, the concentrations of Cd in the edible parts of the four vegetables were reduced with amendment treatments, and the reduction (% of control) was significantly different (P<0.05) among the different treatments. The reduction of Cd in vegetables ranged from 37% to 76% for spinach, and from 34% to 63% for tomato, and from 59% to 76% for Chinese cabbage, and from 61% to 77% for radish, with the lowest for CS treatment and the highest for RS+RM treatment. Although the yield of edible parts of spinach, tomato, Chinese cabbage and radish treated with amendments were increased by 3–36%, 4–13%, 6–25% and 1–31%, respectively (Table 1), the total Cd uptake in edible parts of the four vegetables was still significantly decreased with amendment treatments (data can be calculated using the Cd concentration multiplying by yield of vegetables in Table 1) as there was “dilution effect” of Cd in edible parts of plant. Combined with amendment treatments, Zn application further decreased the Cd concentration in vegetables up to 74–84% of those in unamended treatment.

**Table 1:** The concentration of Cd (C_Cd_, mg Cd kg^−1^ in dry weight) and yield (g plant^−1^ in dry weight) for edible part of spinach, tomato, Chinese cabbage and radish in Cd-contaminated soils (added Cd at 1.5 mg kg^−1^) with different amendments with (+Zn) and without Zn fertilization.

Treatment	Spinach		Tomato		Chinese cabbage		Radish	
	C_Cd_ (mg kg^−1^)	Yield (g plant^−1^)	C_Cd_ (mg kg^−1^)	Yield (g plant^−1^)	C_Cd_ (mg kg^−1^)	Yield (g plant^−1^)	C_Cd_ (mg kg^−1^)	Yield (g plant^−1^)
CK	0.75±0.05 a	0.96±0.03 c	0.35±0.03 a	4.31±0.03 d	0.49±0.03 a	161±1 h	0.56±0.02 a	94±1 d
CK+Zn	0.53±0.03 b	1.11±0.02 b	0.31±0.03 b	4.58±0.06 bc	0.35±0.02 b	164±2 h	0.45±0.02 b	102±1 c
RM	0.40±0.04 c	1.14±0.03 b	0.18±0.02 d	4.61±0.03 bc	0.19±0.02 c	175±1 f	0.19±0.01 d	115±1 b
RM+Zn	0.29±0.03 d	1.21±0.04 ab	0.16±0.02 de	4.69±0.06 b	0.16±0.01 d	191±2 c	0.16±0.01 e	117±1 b
RS	0.41±0.05 c	0.91±0.03 c	0.14±0.02 e	4.51±0.04 c	0.16±0.01 d	179±2 e	0.17±0.01 de	103±1 c
RS+Zn	0.36±0.03 c	0.93±0.04 c	0.13±0.01 e	4.77±0.07 ab	0.12±0.01 e	185±2 d	0.16±0.02 e	108±3 c
RM+RS	0.18±0.02 e	1.25±0.02 a	0.12±0.02 e	4.83±0.05 a	0.12±0.01 e	192±3 c	0.13±0.01 f	117±2 b
RM+RS+Zn	0.12±0.02 f	1.31±0.03 a	0.09±0.02 f	4.89±0.07 a	0.09±0.01 f	201±3 a	0.10±0.01 g	123±1 a
CS	0.47±0.05 bc	0.99±0.03 c	0.23±0.02 c	4.48±0.04 c	0.20±0.02 c	171±2 g	0.22±0.01 c	95±1 d
RM+CS	0.37±0.04 c	1.25±0.04 a	0.16±0.01 de	4.69±0.05 b	0.18±0.01 cd	189±1 c	0.18±0.01 de	113±1 b
RM+CS+Zn	0.26±0.04 d	1.27±0.03 a	0.13±0.02 e	4.62±0.09 bc	0.15±0.01 d	196±1 b	0.15±0.02 e	120±1 a

The application rates in soils were at 0.5% (W/W) for red mud (RM), 0.1% (W/W) for rape straw (RS) and corn straw (CS), and 3 g ZnSO_4_ per square meter, respectively.

Note: Within each column in the same vegetable, mean values ± standard errors with the same letter do not differ significantly at 5% level (P<0.05) according to the Fisher’s least significant test.

### Changes in Cd fractions in soils

**Figure 1:**
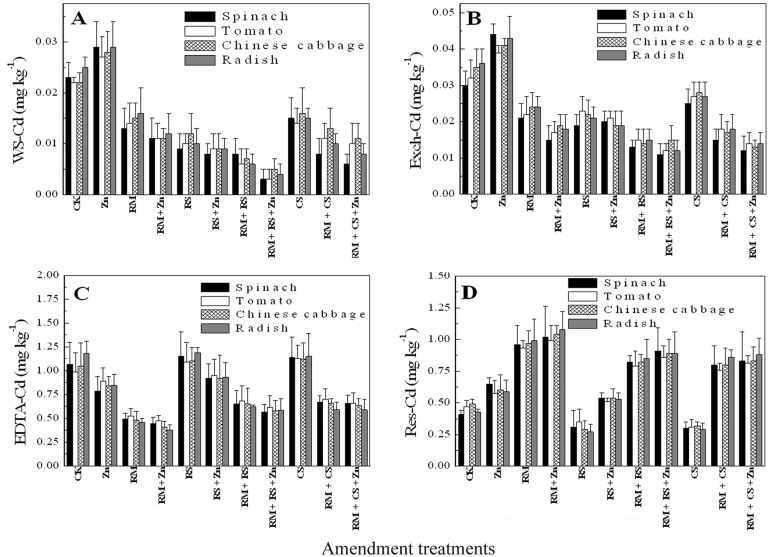
Concentrations (mg Cd kg^−1^ soil) of water soluble (WS-Cd, A), exchangeable (Exch-Cd, B), EDTA extractable (EDTA-Cd, C) and residual Cd (Res-Cd, D) in the soils with and without different amendments after spinach, tomato, Chinese cabbage and radish cultivation.

The concentrations of Cd in different fractions of soils with different treatments after plant harvest were shown in Figure 1. The proportion of Cd in the control (total Cd 1.5 mg kg^−1^) soil were 1.46% (0.023 mg kg^−1^) in the WS-Cd, 2.11% in the Exch-Cd, 67.9% in the EDTA-Cd, and 28.5% in the Res-Cd. The concentrations of WS-Cd and Exch-Cd were significantly lower (P<0.05) in the amended soil than in the unamended soil except Zn treatment (Fig. 1). In general, addition of amendments combined with Zn fertilization to the soil can significantly decrease WS-Cd from 34% (CS) to 84% (RS+RM+Zn) among the four plants. The addition of the RM, RM+CS and RM+RS combined with Zn fertilization also remarkably increased the Res-Cd. As for RS and CS treatments, the EDTA-Cd increased about 5%. Among the fractions, WS-Cd and Exch-Cd were more pronouncedly affected by the treatment of amendments than the other fractions, which suggested that WS-Cd and Exch-Cd were transformed to the non-extractable form in the amended soil.

## Discussion

Accumulative evidence from pot trials [27], [29] and field samples [4], [14], [15], [30] clearly showed that vegetables grown on Cd-contaminated soils results in elevated Cd levels in edible parts of the vegetables, exceeding the maximum allowable limit (0.05 mg kg^−1^ fresh weight) of NFHSC. Our results showed that Cd concentrations of the edible of parts of the spinach, tomato, Chinese cabbage and radish grown in the unamended soil (1.5 mg kg^−1^ Cd exposure) was 0.75 mg kg^−1^ (0.06 mg kg^−1^ fresh weight), 0.35 mg kg^−1^ (0.06 mg kg^−1^ fresh weight), 0.49 mg kg^−1^ (0.053 mg kg^−1^ fresh weight) and 0.56 mg kg^−1^ (0.08 mg kg^−1^ fresh weight), respectively, being 1.06–1.60 fold as high as the NFHSC value (Table 1). When different amendments were added to soil, the Cd concentrations in edible parts of the four vegetables were almost decreased to the values of <0.05 mg kg^−1^ fresh weight. The present results also indicated that distinctive differences in Cd accumulation when comparing one vegetable to another, following the order: spinach (leafy vegetables, Chenopodiaceae) > radish (root vegetables, Cruciferae) > Chinese cabbage (kale vegetables, Cruciferae) > tomato (fruit vegetables, Solanaceae), which is similar with the result in soil with Cd added less than 2 mg kg^−1^ from the study of Yang et al. [31]. The differences in Cd accumulation are probably because the soil-to-plant transfer factor of Cd (TF = M(edible part)/M(soil), M is the Cd concentration in edible part or soil) for the leafy vegetables were higher than those for the non-leafy vegetables [27], [30]. When grown in the unamended soil (1.5 mg kg^−1^ Cd exposure), the TF of edible parts of spinach, radish, Chinese cabbage and tomato was 0.50, 0.37, 0.33 and 0.23, respectively. When applied with the amendments and/or Zn fertilization, the decline in TF of the four vegetables treated with the amendments was significantly different, ranging from 0.08 (RS+RM+Zn) to 0.35 (CK+Zn) for spinach, from 0.07 (RS+RM+Zn) to 0.30 (CK+Zn) for radish, from 0.06 (RS+RM+Zn) to 0.23 (CK+Zn) for Chinese cabbage, and from 0.08 (RS+RM+Zn) to 0.21 (CK+Zn) for tomato. Although all amendments played an important role of decreasing Cd transfer from soil to plant, however, in comparison with other three vegetables, the transfer ability of spinach for Cd were always stronger than others (Table 1). Not only for Cd, spinach could also accumulate other heavy metals (e.g., Ni, Pb and Cu) intensely [31]–[33], for instance, nickel level in spinach shoot was found to be 1.5–4.9 fold as high as those in other six plant species (including tomato and cabbage) in high-pH soil [33]. These results might be related to the characteristic of leafy vegetables, easy uptake/translocation of heavy metals from soil to shoots by passive uptake - transpiration based on bigger surface area of plant leaves and stomatal aperture [34]. Generally, accumulation for heavy metals of different parts of plant is in the order of root > shoot > fruit, which might be the reason that radish (root vegetables) could accumulate more Cd than Chinese cabbage (kale vegetables) and tomato (fruit vegetables).

Previous studies showed that addition of red mud [10]–[12], [14], [15], [35] and plant materials [36], [37] to Cd-contaminated and acidic soils could effectively reduce Cd bioavailability in soils. However, little information was available about the addition of red mud and/or plant materials to mildly Cd-contaminated and calcareous soils (high pH) under field conditions. Results from the present study showed that Cd concentration and uptake in edible parts of the four vegetables treated with RM, RS and CS under mildly Cd-contaminated and calcareous soils was significantly (P<0.05) reduced. The reason might be because the RM, RS and CS application markedly reduced Cd mobility in the Cd-contaminated soil. As shown in Fig. 1, the WS-Cd and Exch-Cd in the amended soils averagely decreased from 26% (CS) to 70% (RS+RM) except Zn treatment, while the Res-Cd averagely increased from 35% (RM+CS) to 108% (RM) among RM, RM+CS and RM+RS treatment, and the EDTA-Cd averagely increased at 5% for RS and CS treatments, suggesting that the addition of RM could lead to Cd transformation from WS-Cd and Exch-Cd to Res-Cd, while the addition of CS and RS could lead to Cd transformation from WS-Cd and Exch-Cd to EDTA-Cd. Furthermore, among the amendments RS+RM was the most effective, with the greatest reductions in WS-Cd (70%) and Exch-Cd (57%). Yang et al. [38] also showed that RS and nano-treated RM were the two best amendments in decreasing Exch-Cd in alkaline soil and total Cd in cucumber plants. The reason might be ascribed mainly to different mechanisms of bindings of Cd between these amendments. In the present study, th e application of RM (0.5%, W/W), RS (0.1%, W/W) and CS (0.1%, W/W) to the calcareous DZ soil (pH 8.9) had no obvious increase on soil pH (data not shown). The transformation of WS-Cd and Exch-Cd to Res-Cd was probably due to the specific sorption of Cd by Fe and Al oxides in RM, while the transformation of WS-Cd and Exch-Cd to EDTA-Cd was probably due to the complexation with RS or CS. Luo et al. [39] further investigated the sorption mechanism of cadmium on red mud as same as used in the study using batch sorption experiments, sequential extraction analysis and X-ray absorption near edge structure (XANES) spectroscopy and supplied evidence of the formation of inner-sphere complexes of Cd similar to XCdOH (X represents surface groups on red mud) on the red mud surfaces although outer-sphere complexes of Cd were the primary species.

Some studies revealed that plant materials could be sorbent materials for Cd due to the tendency of Cd to form stable complexes with organic ligands [36], [40]. Wu et al. [41] found that the Cd concentrations in grains of rice by rotation with rape were decreased approximately by 46–80% of those for rice cultivation only, the decreasing of WS-Cd plus Exch-Cd and increasing of Org-Cd was might be related to the abundance of organic material secreted from rape roots in soil. Harada et al. [17] also found that Cd stress could result in a 3-fold increase in total thiols mainly contributing to synthesis of cysteine, glutathione and phytochelatins in *Arabidopsis*. In the present study, an obvious decrease of WS-Cd in soil was displayed with the addition of RS (Fig. 1), which might be ascribed to the high affinity for Cd induced by sulfur compounds (thiol) in rape straw. However, for CS, a relative lower affinity for Cd could be resulted from (semi)cellulose as main components in straw [42].

**Figure 2:**
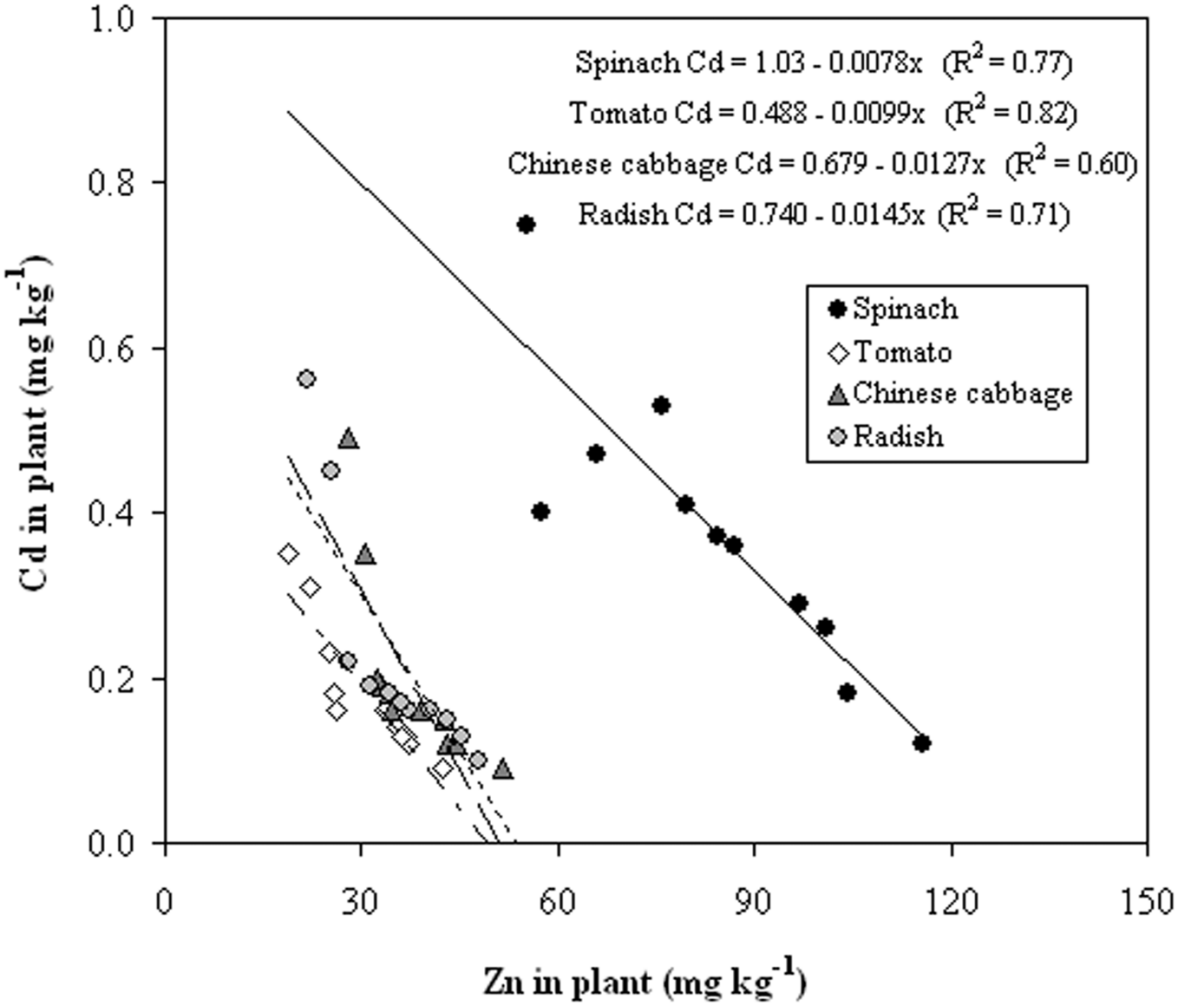
Concentration of Cd in edible parts of spinach, tomato, Chinese cabbage and radish as a function of the Zn concentration in these plants in Cd-contaminated soils (1.5 mg added Cd kg^−1^ soil) with different amendments with and without Zn fertilization.

Results from the present study indicated that Cd concentrations in the edible parts of vegetables were significantly lower for amendment treatments with Zn fertilization than those with amendments only (Table 1). Furthermore, the results (Fig. 2) also showed that there were significant negative correlations between concentrations of Cd and Zn in edible parts of vegetables with R^2^≥0.60 (P<0.01, n = 11), and supplied the evidence of Zn antagonistic effect on Cd uptake by plants in the calcareous soils with amendments. Oliver et al. [22] also found Zn fertilization markedly reduced Cd concentration in wheat grain in areas where it was marginal to severe Zn deficiency in South Australia. These results suggested that Zn fertilization could be a practical measure combined with soil amendments to decrease Cd concentration in plants in soils where the Zn availability is low.

Recently, there is evidence that RM combined with other amendment, such as gravel sludge (GS), a waste product of the gravel industry, had higher long-term efficiency in immobilizing Cd than only RM or GS treatment under field condition [15]. Similarly, our results provided clearly evidence for a synergistic interaction between RM and RS leading to highly significant (P<0.01) reductions in the Cd accumulation of edible parts of the four vegetables. Considering all of the above-mentioned facts, the RS+RM is suggested to act as an efficient, economic and practical measure for mildly Cd-contaminated and calcareous soil, best in combination with Zn fertilization if soils with low Zn availability.

## Conclusions

This study clearly demonstrated that water soluble and exchangeable Cd in soils was significantly decreased by red mud, rape and corn straw, which resulted in significant decrease by about 34% to 77% in Cd concentration in vegetables in Cd-contaminated and calcareous soils. Combined with the amendments, Zn fertilization further decreased Cd concentration in the edible part of vegetables up to 74% to 84%. The effect of rape and corn straw could be ascribed to formation of stable complexes with organic ligands while red mud to specific sorption of Cd. Also cruciferous rape straw was more effective than gramineous corn straw. In all treatments, rape straw plus red mud combined with Zn fertilization was most effective in decreasing Cd phytoavailability in soils, and it is potential to be an efficient and cost-effective measure to ensure food safety for vegetable production in mildly Cd-contaminated and calcareous soils.
